# A Structural Insight into Major Groove Directed Binding of Nitrosourea Derivative Nimustine with DNA: A Spectroscopic Study

**DOI:** 10.1371/journal.pone.0104115

**Published:** 2014-08-07

**Authors:** Shweta Agarwal, Deepak Kumar Jangir, Ranjana Mehrotra, Neelam Lohani, M. R. Rajeswari

**Affiliations:** 1 Quantum Optics and Photon Physics, CSIR-National Physical Laboratory, New Delhi, India; 2 Department of Biochemistry, All India Institute of Medical Sciences, New Delhi, India; National Institute for Medical Research, Medical Research Council, London, United Kingdom

## Abstract

Nitrosourea therapeutics occupies a definite place in cancer therapy but its exact mechanism of action has yet to be established. Nimustine, a chloroethyl nitrosourea derivative, is used to treat various types of malignancy including gliomas. The present work focuses on the understanding of nimustine interaction with DNA to delineate its mechanism at molecular level. Attenuated total reflection-Fourier transform infrared (ATR-FTIR) has been used to determine the binding sites of nimustine on DNA. Circular dichroism (CD) spectroscopy has been used to confirm conformational variations in DNA molecule upon nimustine-DNA interaction. Thermodynamic parameters of nimustine-DNA reaction have been calculated by isothermal titration calorimetry. Results of the present study demonstrate that nimustine is not a simple alkylating agent rather it causes major grove-directed-alkylation. Spectroscopic data suggest binding of nimustine with nitrogenous bases guanine (C6 = O6) and thymine (C4 = O4) in DNA major groove. CD spectra of nimustine-DNA complexes point toward the perturbation of native B-conformation of DNA and its partial transition into C-form. Thermodynamically, nimustine-DNA interaction is an entropy driven endothermic reaction, which suggests hydrophobic interaction of nimustine in DNA-major groove pocket. Spectral results suggest base binding and local conformational changes in DNA upon nimustine interaction. Investigation of drug-DNA interaction is an essential part of rational drug designing that also provides information about the drug’s action at molecular level. Results, demonstrated here, may contribute in the development of new nitrosourea therapeutics with better efficacy and fewer side effects.

## Introduction

**Figure 1:**
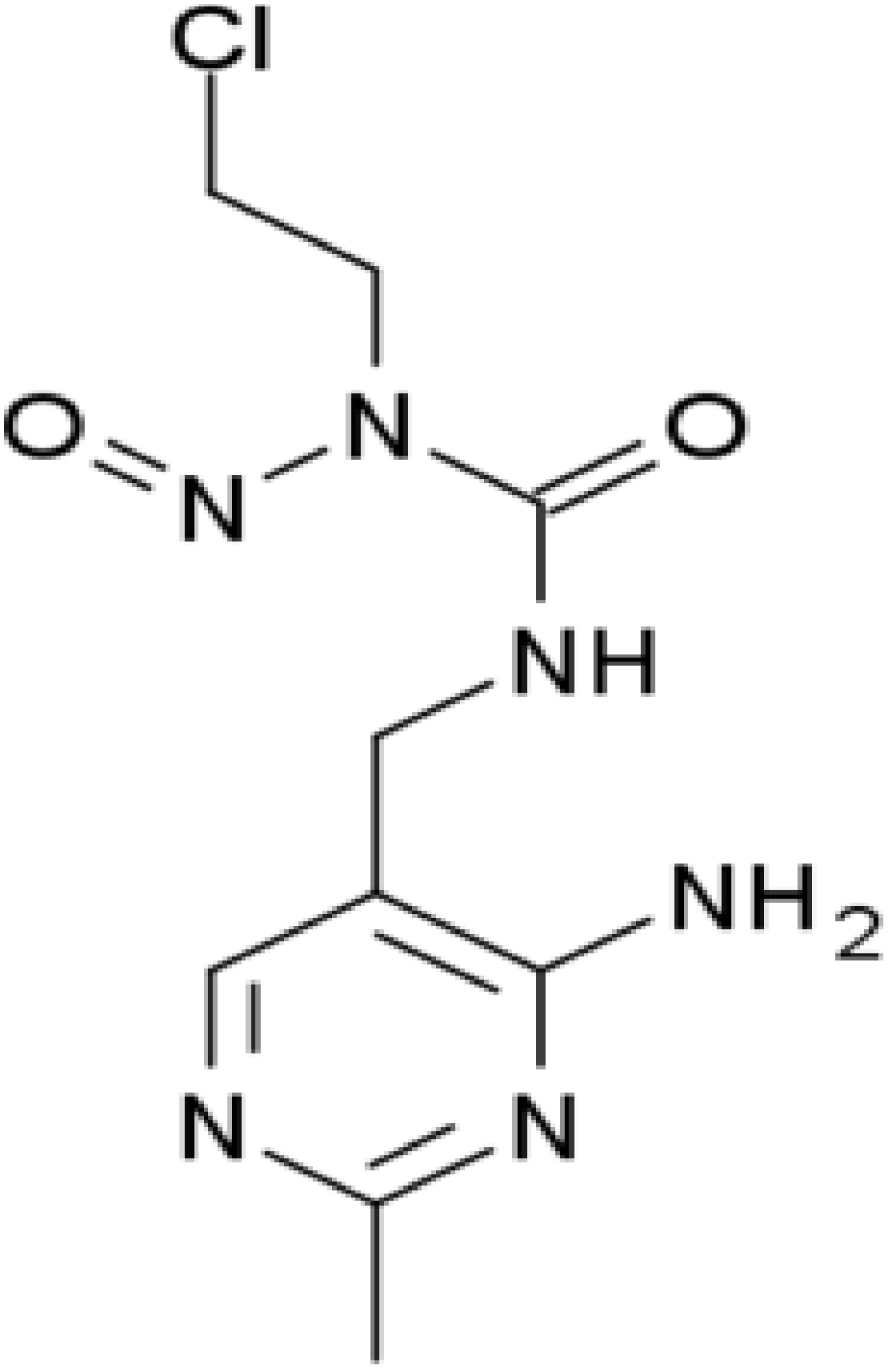
Chemical structure of nimustine.

Most of the anticancer agents, currently used, have DNA and auxiliary processes as their main target in the cell [1]. Therefore, a comprehensive understanding on the physical/chemical interactions between DNA and small molecules (drug) becomes vital in an effort to search potential drug candidates for targeted therapy [2]. Results of such investigations can suggest the modification of the drug molecule in a way that produces fewer side effects and more efficiency [3]. Interaction studies can improve the understanding on the binding mechanism of the drug with its target molecule. Further, such investigations can offer details on the moieties that are involved in the interaction [3]. Alkylating agents comprise a major class of therapeutics, which are used in the treatment of different types of cancer [4]. Nimustine or ACNU [(1-(4-amino-2-methyl-5-pyrimidynyl) methyl-3-(2-chloroethyl)-3-nitrosourea hydrochloride)] (Figure 1), one of the derivatives of nitrosourea, is used as an alkylating anticancer drug [5]. It is a cell-cycle phase nonspecific antineoplastic agent, mainly used for the treatment of malignant gliomas (brain/spine tumor) [5]. Nimustine, in addition to radiation therapy, provides a major therapeutic option for the high-grade gliomas [6]. Nimustine is soluble both in both water and methanol, representing its affinity to lipid bilayer membrane, which enables it to cross the blood-brain-barrier for the chemotherapy of gliomas [7]. Despite the availability of detailed structural/chemical knowledge of the alkylating agents in the literature, there remain deficits in the understanding of nimustine-DNA interaction. Experimental evidence has indicated the correlation of *O^6^-methylguanine-DNA methyl transferase* (MGMT) gene expression level with cellular response to nimustine [8]. Mineura *et al* carried out *in vitro* interaction studies between nitrosoureas (involving nimustine) and Hind III digested cellular DNA fragments. Subsequently, on piperidine hydrolysis, they found that nimustine makes scission in DNA fragments corresponding to the location of guanine [9]. The formation of double stranded breaks (DSB) in response to nimustine interaction with DNA has already been reported [10]. Further, observations on specific components activity of DSB-repair pathway suggest that low activity of DNA ligase IV increases cell lethality towards nimustine [10]. Despite the immense importance and direct relevance of nimustine-DNA interactions, the underlying molecular mechanism of nimustine interaction with DNA has not been explored so far.

Significant developments have been made over the past few years in the area of drug-DNA interactions using different biophysical methods and spectroscopic techniques [11], [12]. Attenuated total reflection -Fourier transform infrared (ATR-FTIR) spectroscopy has emerged as an efficient tool in providing the whole information on biomolecule structure and their complexes. It is a fast technique, which provides results in a single snapshot [13]. ATR-FTIR can be applied on short oligonucleotide to full length DNA and can be used for liquid samples at physiological mimic conditions. Recent advances in ATR-FTIR spectroscopy have enabled its extensive use in investigation of nucleic acid binding properties of small ligands and drugs [13]–[16]. In addition, circular dichroism (CD) spectroscopy is highly sensitive technique to determine conformational transition in biomolecules. It can even distinguish sub-conformational isomerization between distinct conformers [17], [18]. Another parameter of paramount importance is thermodynamic profiling (enthalpy, entropy and Gibb’s free energy) of drug-DNA complexes [1], [19]. Isothermal titration calorimetry (ITC) can reveal information on the nature of forces that drive the complex formation. ITC is particularly important for determining the binding affinity, enthalpy, entropy and reaction stoichiometry with high accuracy [19], [20].

In the present study, we have utilized the potential of ATR-FTIR, CD spectroscopic techniques and ITC to study intimate binding properties of nimustine to DNA. The structural evidence, offered here, delivers essential information on the DNA interaction properties of nimustine. This knowledge may provide inputs for the formulation of new nitrosourea derivatives with anti-cancer potential.

## Materials and Methods

### Sample Preparation

Nimustine (M.W- 272.69) and highly polymerized type I calf thymus DNA were procured from Sigma Aldrich chemicals, USA. Ratio of the absorbance of DNA at 260 nm (A_260_) and 280 nm (A_280_) was used to determine the purity of DNA. The calculated ratio of (A_260_)/(A_280_) in DNA sample was found to be 1.81, suggesting the sufficient purity of DNA [21]. Other reagents and chemicals utilized in this investigation were of analytical grade. Deionized ultra pure water (Scholar-UV Nex UP 1000 system) having resistance of 18.2 MΏ was used for the preparation of buffer solution and nimustine drug solutions. Stock solution of DNA sodium salt was prepared in 10 mM tris-HCl buffer (pH 7.4). This solution was placed at 8°C for 24 hour in conjunction with stirring at regular intervals for maintaining the homogeneity of DNA solution. Final concentration of DNA stock solution was measured spectrophotometrically using molar extinction coefficient of 6600 cm^−1^ M^−1^ [22]. The final concentration of DNA stock solution was 42 mM due to molarity of phosphate group.

### ATR-FTIR Spectroscopic Measurement

**Figure 2:**
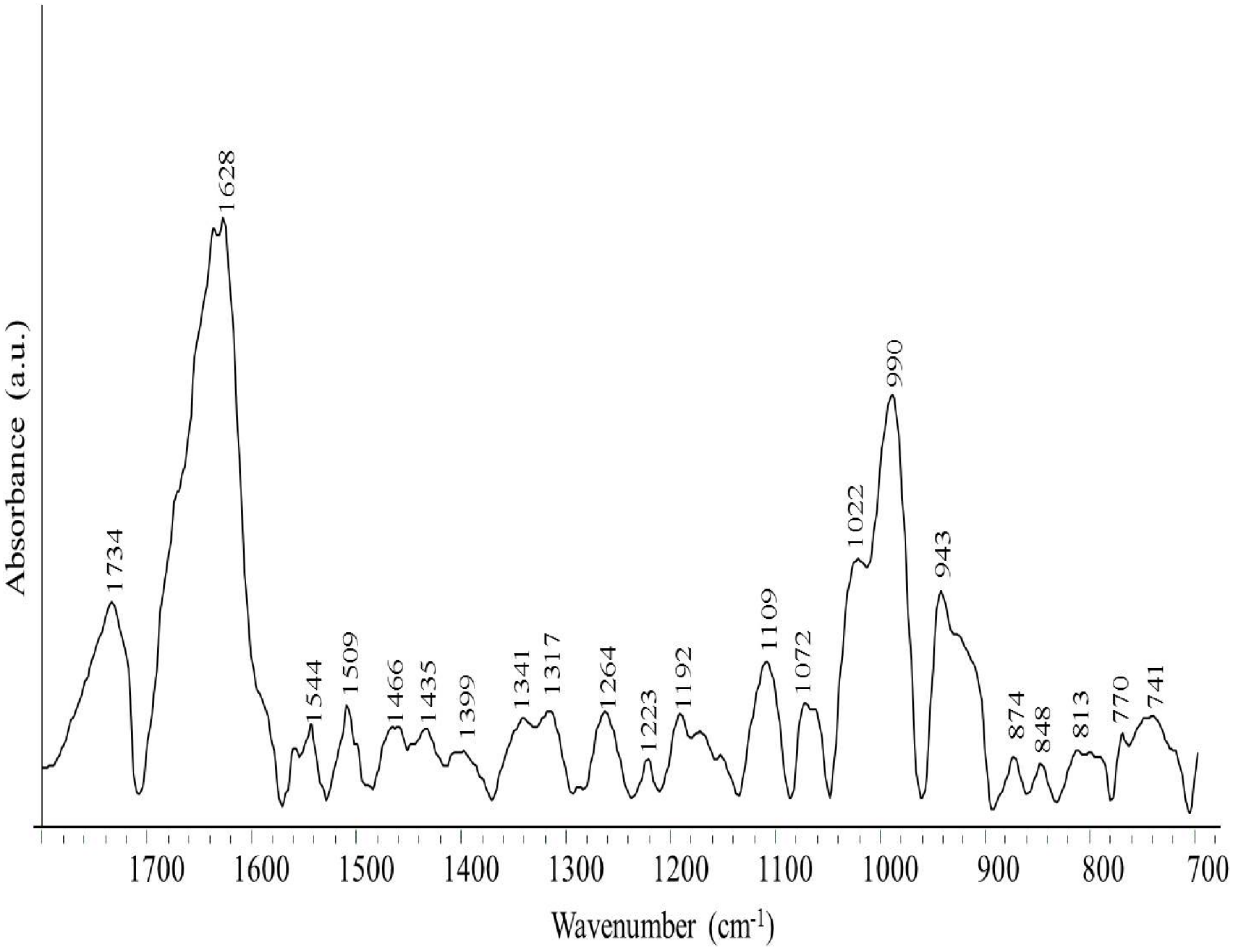
FTIR spectrum of free nimustine in the region of 1800 cm^−1^ to 700 cm cm^−1^.

For studying nimustine-DNA interaction, nimustine solution of varying concentration was added separately dropwise into DNA solution of constant concentration (42 mM) to attain 1/60, 1/40 and 1/20 molar ratios (r). This is followed by continous vortexing for 15 minutes and incubation at room temperature for two hour to ensure the complexation of nimustine with DNA. FTIR spectral measurements of free calf thymus DNA and nimustine-DNA complexes were recorded on Varian-660-IR spectrophotometer equipped with KBr beam splitter and deuterated triglycine sulphate (DTGS) detector. Continuous purging of dry nitrogen gas was performed to remove water vapors from sample chamber. For the sampling in ATR mode, Miracle (PIKE) ZnSe-micro horizontal attenuated total internal reflection (HATR) assembly was used. Ambient humidity of 46% RH was maintained during the experiments. Two hundred fifty six interferograms with a resolution of 2 cm^−1^ were collected in the spectral range of 2400–700 cm^−1^. Before the recording of each measurement, background atmospheric spectrum was collected. No data treatment was performed except multiple baseline correction, water subtraction and normalization for DNA band at 968 cm^−1^. To execute water subtraction, a spectrum of tris buffer was recorded and then subtracted from the spectra of free DNA and nimustine-DNA complexes. An acceptable water subtraction was achieved when the intensity of water combination band at about 2200 cm^−1^ became zero in all the spectra collected [23]. Infrared spectrum of free nimustine was also recorded (Figure 2) and subtracted from the nimustine-DNA complexes spectra. This was done to make sure that observed spectral variations in DNA are due to nimustine binding.

### CD Spectroscopic Measurement

CD spectral measurements were carried out on Applied Photophysics (Chirascan) spectrophotometer. Spectra were collected in the far UV range (200 nm–320 nm) using quartz cuvette having pathlength of 1 mm. Spectral collection was done at room temperature after two hours incubation of nimustine with DNA. For each sample, five scans were recorded with a scanning speed of 1 nm/sec and then averaged. To perform subtraction, spectrum of buffer was subtracted from the spectra of free DNA and nimustine-DNA complexes. CD investigations were performed using nimustine solution of various concentration in the range of 0.041–0.125 mM with constant DNA concentration of 2.5 mM to attain 1/60, 1/40 and 1/20 molar ratios (r).

### ITC Measurement

ITC was performed using NANO-ITC (isothermal titration calorimeter-USA) system at 25°C temperature. Total twenty serial injections of nimustine (1.5 mM) were added at the interval of 200 seconds to calf thymus DNA (5×10^−3^ mM). A control experiment was carried out to calculate the heat of dilution for DNA into buffer (pH-7.4). The net enthalpy-entropy changes for nimustine-DNA interaction was determined by subtracting the corresponding heat of dilution derived from the injection of same amount of nimustine into buffer alone.

## Results and Discussion

### FTIR Spectral Outcome

#### DNA Base Binding

**Figure 3:**
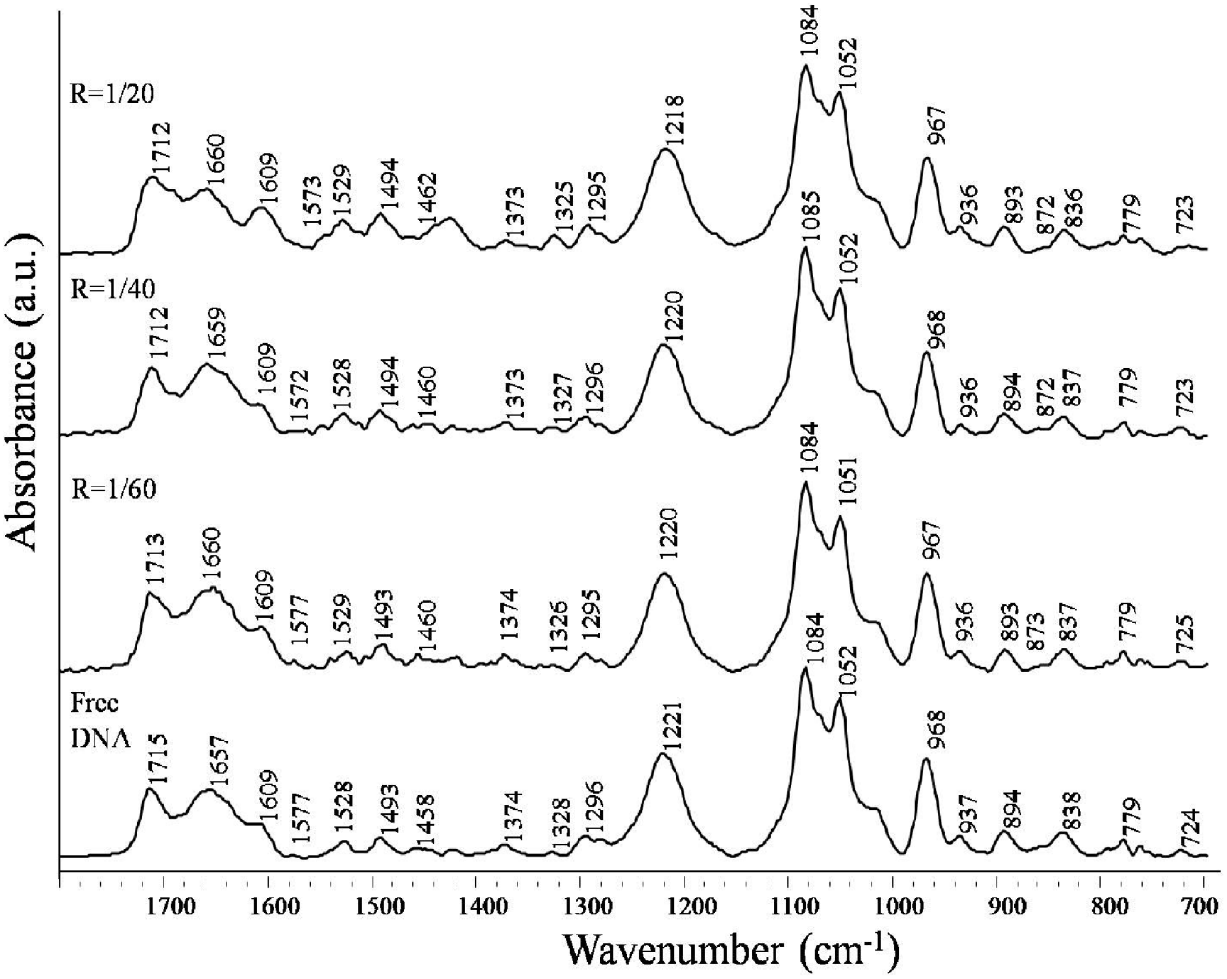
FTIR spectra of free DNA and nimustine-DNA complexes. FTIR spectra of free calf thymus DNA and its complexes with nitrosourea derivative nimustine at different molar ratios were collected in the region of 1800 cm^−1^ to 700 cm cm^−1^.

The infrared spectral features observed in the spectrum of free calf thymus DNA and nimustine-DNA complexes are shown in Figure 3. Stretching vibrations due to deoxyribose sugar, phosphate (PO2- symmetric and asymmetric) and nitrogenous bases (C = O, C = N) of DNA lie in the region of 1800–700 cm^−1^. The infrared band at 1715 cm^−1^ is assigned to guanine (G) due to in-plane stretching vibrations of C6 = O6 bonds [13]–[15], [24]. The band at 1657 cm^−1^ is attributed primarily to thymine (T) stretching vibrations of C4 = O4 bonds [13]–[15], [24]. The bands at 1609 cm^−1^ and 1493 cm^−1^ appear due to the ring stretching vibrations of adenine (C = N) and cytosine (C = C) respectively [13]–[15], [24].

**Figure 4:**
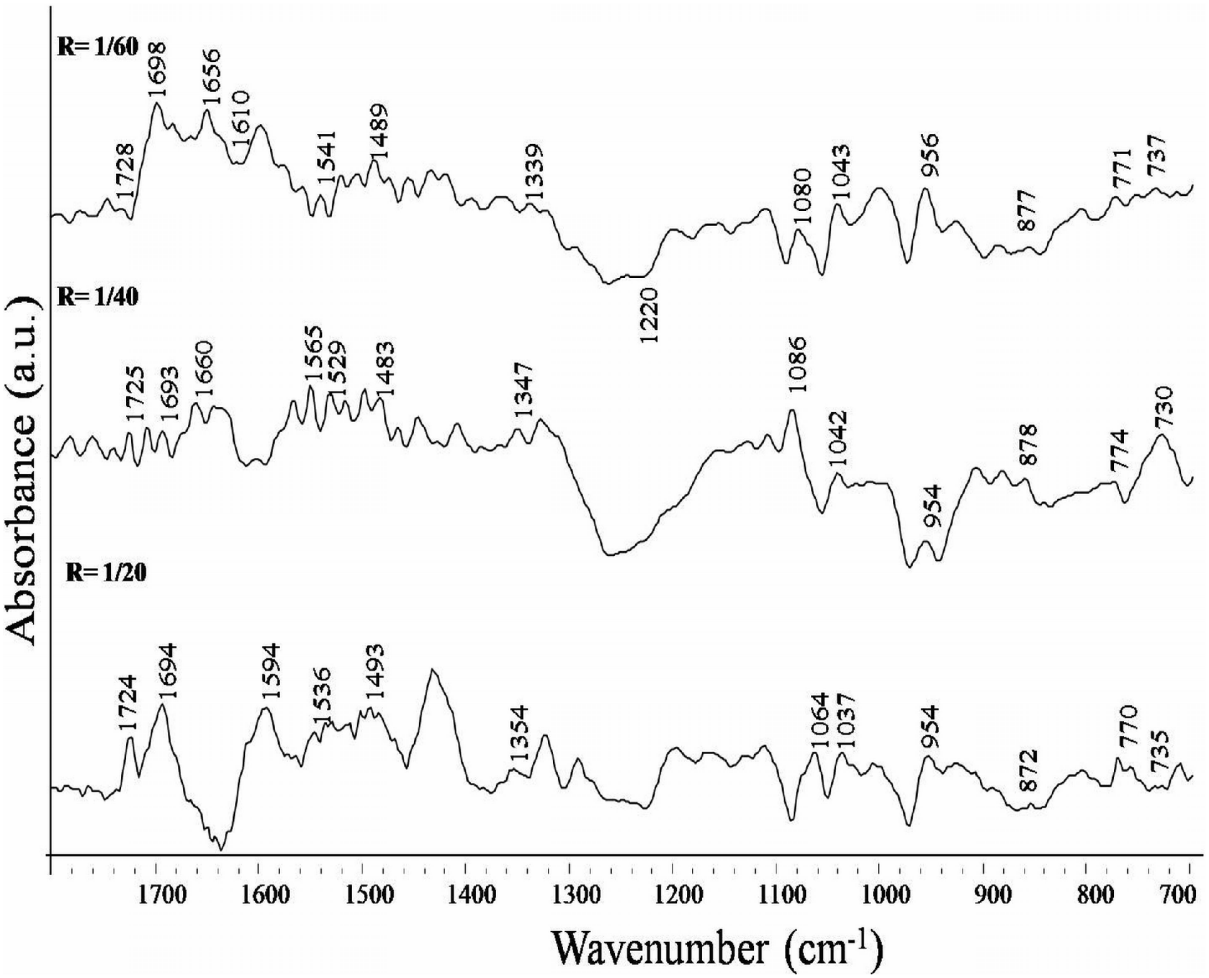
Difference spectra of nimustine-DNA complexes in the region of 1800 cm cm^−1^ to 700 cm cm^−1^. {Difference spectra = [(DNA solution+nimustine solution)–(DNA solution)]}.

**Figure 5:**
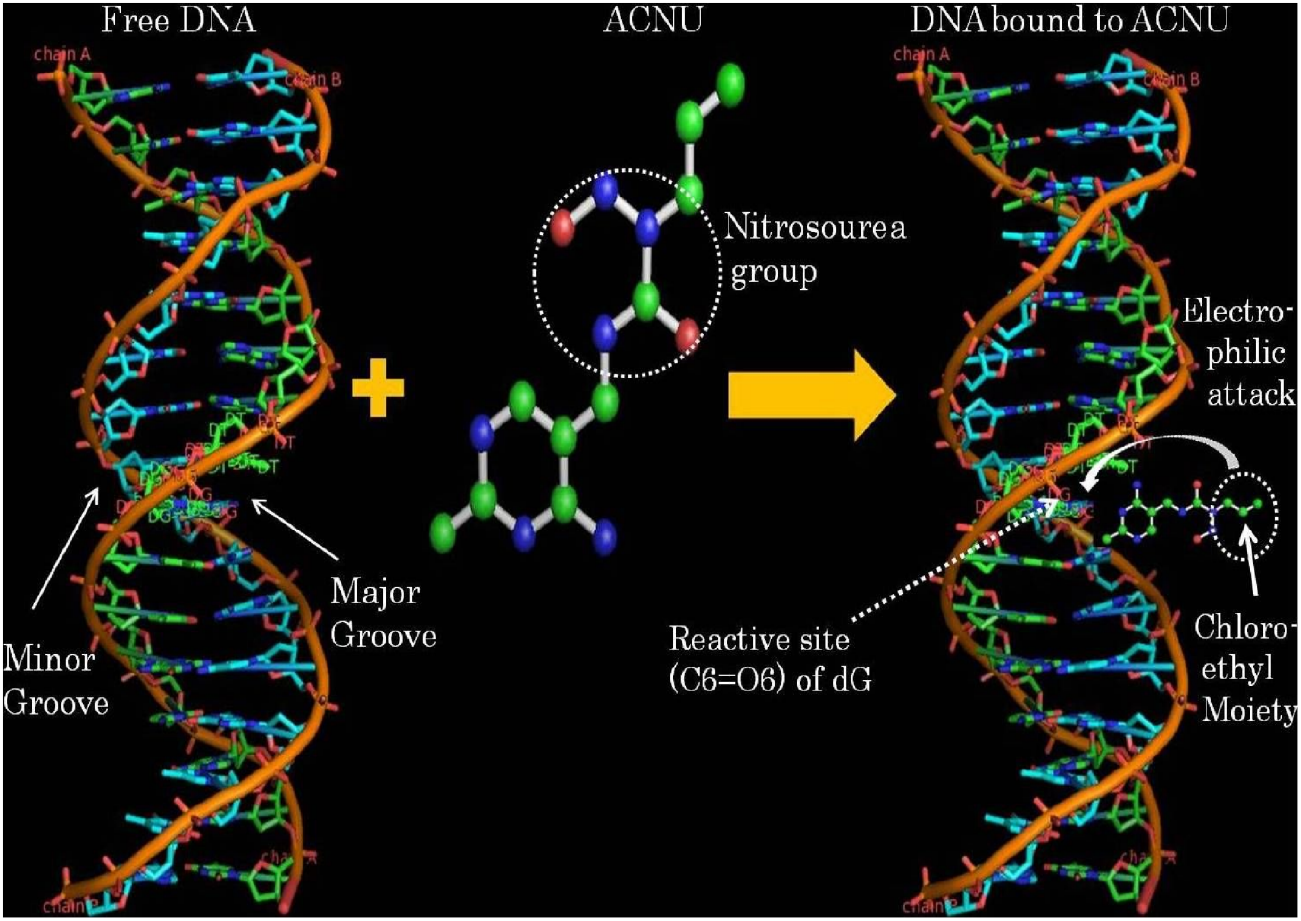
The proposed model for binding of nimustine to DNA.

**Figure 6:**
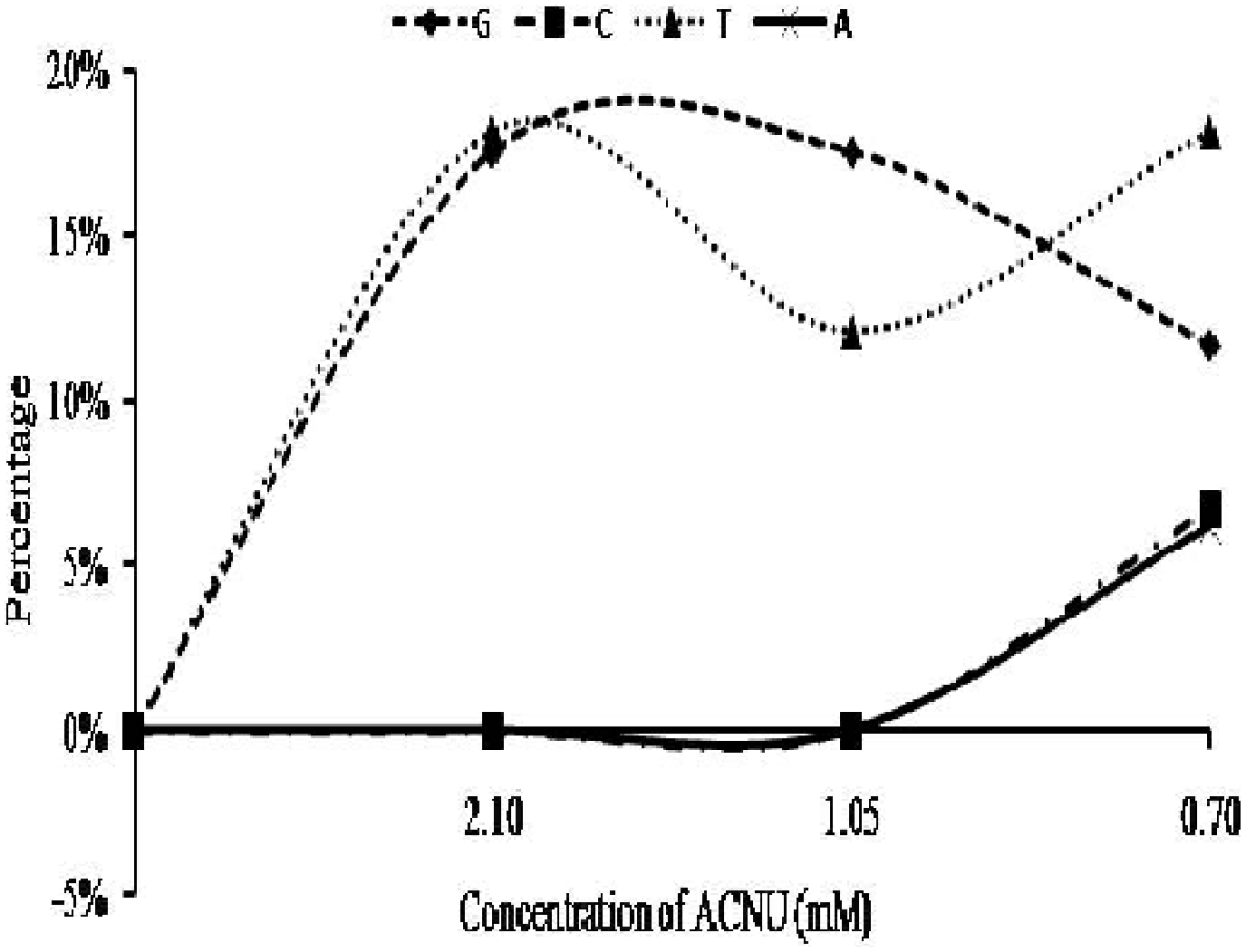
Percentage effect of nimustine on DNA major groove. Percentage effect of nimustine on DNA nitrogenous base guanine (G- C6 = O6) 1715 cm cm^−1^ and thymine (T- C4 = O4) 1657 cm cm^−1^) and other bases of DNA adenine (A) and cytosine (C) was observed as a function of nimustine concentration.

Infrared band observed in the spectrum of free DNA at 1715 cm^−1^ (guanine) shows downshift of 3 cm^−1^ (from 1715 cm^−1^ to 1712 cm^−1^) in nimustine-DNA complexes. Similarly, up-shift of 3 cm^−1^ is observed in infrared band assigned to thymine (from 1657 cm^−1^ to 1660 cm^−1^). Band at 1493 cm^−1^ (cytosine) shows 1 cm^−1^ up-shift upon nimustine interaction with DNA. Shifts in band position for the nitrogenous bases of DNA are also found accompanied by the changes in intensity at all molar ratios of nimustine-DNA complexes. Positive bands at 1724 cm^−1^ and 1652 cm^−1^ are observed in the difference spectra of nimustine-DNA complexes [(DNA solution+nimustine solution)–DNA solution] (Figure 4). These bands specify infrared hyperchroism for the stretching vibrations of guanine and thymine respectively [13]–[15]. Minor increase in intensity of cytosine is also evident by positive infrared features at around 1493 cm^−1^. No appreciable change in intensity and position of adenine band at 1609 cm^−1^ is noticed in nimustine-DNA complexes. Deviations in intensity and shift in the infrared bands associated with guanine and thymine suggest direct interaction of nimustine with the moieties of these heterocyclic nitrogenous bases [13]–[15]. Furthermore, groups C6 = O6 (guanine) and C4 = O4 (thymine) are located in major groove of DNA. Therefore, spectral variations at 1715 cm^−1^ and 1657 cm^−1^ augment the possibility of nimustine mechanism via ‘major groove-directed-alkylation’ [25]. The plausible explanation of these spectral observations can be that nimustine is first positioned within DNA major groove and then performs alkylation via transfer of chloroethyl moiety (from nimustine) to O6 position of guanine (Figure 5). Results are in corroboration with the fact that “most of the alkylating agents are major groove binder” [26]. Phenomenon of groove binding followed by alkylation has also been observed in the case of altromycin B [27] and anthramycin [28]. Along with the spectral changes (shifts and intensity change), percent effect of nimustine binding on four bands representative of reactive sites guanine C6 = O6, thymine C4 = O4 (located in major groove), cytosine and adenine is shown in Figure 6.

#### Phosphate-Sugar Backbone Binding

In the spectrum of free calf thymus DNA (Figure 3), infrared bands appeared at 1221 cm^−1^ and 1084 cm^−1^ are due to phosphate asymmetric and symmetric stretching vibrations respectively [13]–[15], [24]. Downward shift of 3 cm^−1^ is observed in phosphate asymmetric stretching vibrations band at 1221 cm^−1^ at highest molar ratio (1/20). No appreciable shift is noticed in the band of phosphate symmetric vibrations at 1084 cm^−1^. In the difference spectra (Figure 4), positive band at 1086 cm^−1^ and negative band at 1220 cm^−1^ are observed, which indicate change in intensity of phosphate stretching vibrations upon drug interaction. Deoxyribose sugar vibrations due to C = O and C-C stretching are denoted by infrared bands at 1052 cm^−1^ and 968 cm^−1^ in the spectrum of free calf thymus DNA [13]–[15], [24]. No significant shift is observed in these bands in the complexes however; both of these bands show minor infrared hyperchroism (positive bands at 1043 cm^−1^ and 956 cm^−1^ in difference spectra). All of these observations suggest slight external binding of nimustine with phosphate-sugar backbone of DNA double helix [13]–[15], [24]. Bands at 1374 cm^−1^, 1296 cm^−1^, 779 cm^−1^ and 724 cm^−1^ are assigned to sugar conformations [13]–[15], [24] and show negligible shift when the nimustine-DNA interaction takes place. In the difference spectra of nimustine-DNA complexes, positive features are observed at about 1347 cm^−1^, 770 cm^−1^ and 730 cm^−1^ due to the increase in intensity of sugar vibrations. Spectral changes observed for the sugar conformations, deoxy ribose-phosphodiester chain vibrations and sugar-phosphate backbone stretching vibrations suggest fine external binding of nimustine with DNA [13]–[15].

#### DNA Conformation

Infrared band, emerges due to S-C_2_ endo/anti sugar pucker-phosphodiester stretching vibrations at 838 cm^−1^, is regarded to be marker of B-conformation of DNA (Figure 3) [24], [29]. Moreover, infrared bands at 1221 cm^−1^ (antisymmetric PO2 stretching) and 894 cm^−1^ (deoxyribose ring stretching) are also characteristic feature of B-DNA [24], [29]. In addition, conformation of C-N glycosidic linkage (infrared band at 1458 cm^−1^) is also responsible for the maintenance of DNA in B-form [24], [29]. Upon nimustine complexation with DNA, band at 1221 cm^−1^ is shifted to lower frequency (1218 cm^−1^), which suggests a decrease in DNA hydration state [30]. The infrared band at 1458 cm^−1^ (glycosidic bond) is shifted to 1462 cm^−1^ after nimustine-DNA interaction. Besides this, there is an emergence of new band at 872 cm^−1^, which is considered a characteristic feature of DNA in C-conformation [30]. Reduction in hydration state around phosphate group of DNA with the appearance of new band at 872 cm^−1^ indicates the transition of duplex from B to C-form [30]. Nevertheless, this transition occurs at local level, as evident by the presence of other prominent DNA B-form markers (1220 cm^−1^ and 837 cm^−1^) in nimustine-DNA spectra. These changes confirm that DNA remains globally in B-form.

### CD Spectral Outcome

#### Affirmation of DNA Conformational changes

**Figure 7:**
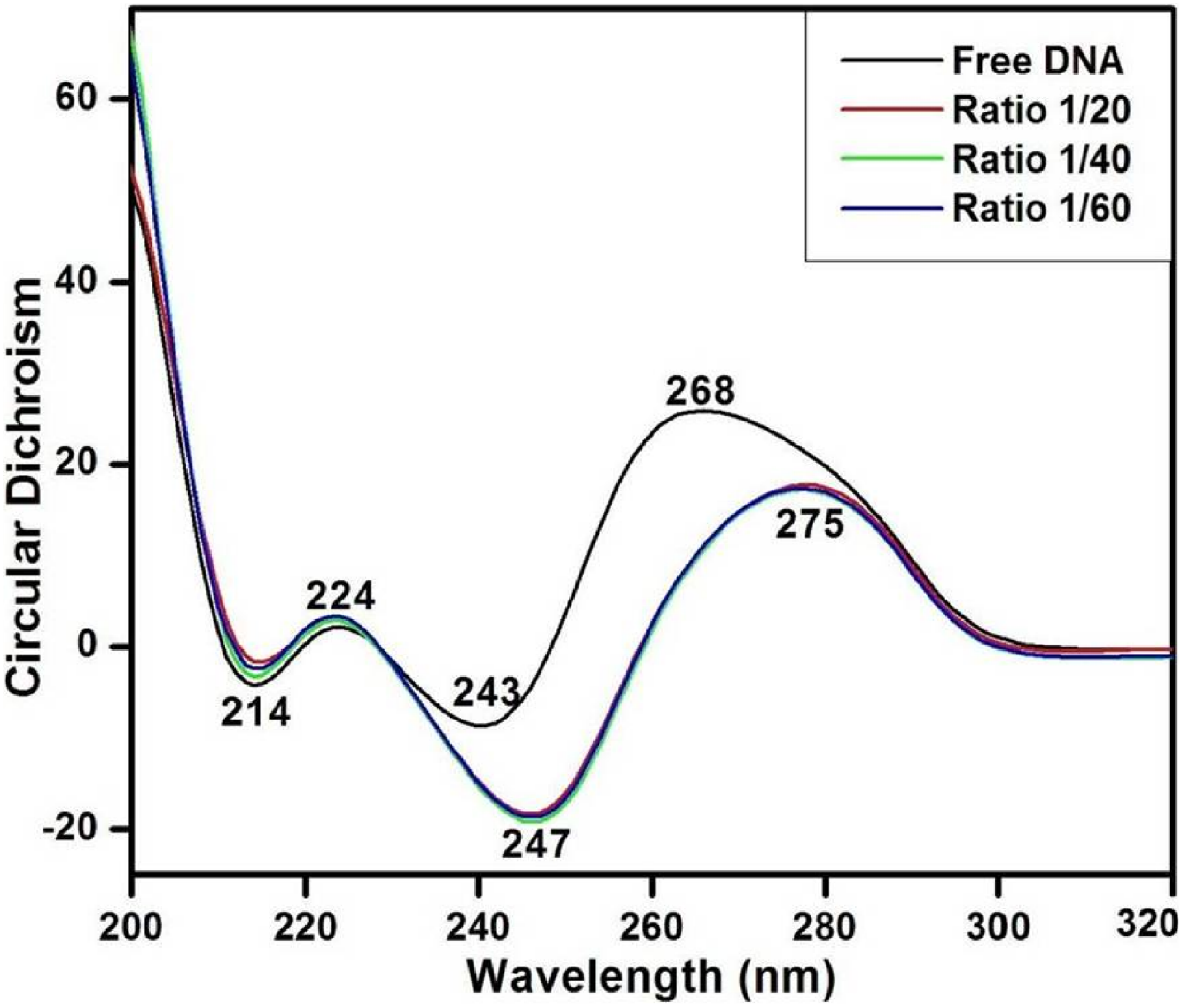
Circular dichroism spectra of free DNA and nimustine -DNA complexes at different molar ratios.

Figure 7 shows the CD spectra of free calf thymus DNA and its complexes with nimustine. Asymmetrical glycosidic bond and specific right-handed helical arrangement of B-DNA give a typical CD spectrum that comprises two positive and two negative ellipticitical components: 268 nm (positive), 243 nm (negative), 224 nm (positive) and 214 nm (negative). Band at 268 nm arises due to the stacking interaction between the nitrogenous bases while the band at 243 nm is attributed to the right-handedness of B-DNA [31]–[33]. Manifestation of bands at 214 nm and 224 nm is due to β-N-glycosidic linkage (present between nitrogenous base and deoxyribose sugar) and hydrogen bonds occurring between the nitrogenous bases of opposite strands respectively [31]–[33]. Alteration in band position as well as in intensity of these spectral bands is due to the corresponding conformational transitions in duplex DNA allied to its interaction with drug. Upon the addition of nimustine, the band at 268 nm (assigned to base stacking) shows bathochromic shift (red shift) of 7 nm along with decrease in positive ellipticity (32%). Furthermore, negative band (243 nm) attributed to helicity shows red shift of 4 nm with reduction in ellipticity (44%) at all molar ratios. Red shift and decrease in molar ellipticity at these bands (268 nm and 243 nm) suggest the distortion in native conformation of B-DNA due to nimustine interaction [33]. A loss of 268 nm CD intensity along with red shift has been correlated with small change in number of base pair per turn in DNA helix [30], [34]–[36] and reflects the increase in DNA winding angle [37]. These spectral variations show the presence of some C DNA features in native conformation of DNA upon nimustine interaction. Moreover, reduction in 243 nm CD band is considered a key marker of C-form of DNA [30], [34]–[36]. Therefore, altogether, the presence of these spectral features augment a possibility of the perturbation of DNA conformation from B (10.4 base pair/helical turn) to C-form (∼9.4 base pair/helical turn) [30], [35], [38]. However, it seems that the perturbation in DNA conformation is limited to few base pairs. When complete B to C transition occurs, then the CD band at 243 nm shows about 66% decrease in its intensity. However, not much decrease in the concerned band is observed. Hence, there are possibilities of the formation of an intermediate form of DNA having features of both B and C conformation. Similar results have been observed in the case of cationic lipid [30] and neutral lipid binding with DNA [39] that have been ascribed to a non-cooperative augment in DNA winding angle due to change in base pair per turn from 10.4 to 9.8. Moreover, increase in winding angle (or decrease in propeller twist) causes widening in DNA groove [40] that enables proper positioning of small ligands in the groove pocket. This suggests that nimustine is partially positioned within the major groove and modulates accessibility for alkylation of nitrogenous base via transfer of chloroethyl moiety from drug to O6 position of guanine. This is in accordance with FTIR results that signify the interaction of nimustine with guanine (C6 = O6) and thymine (C4 = O4), representative of major groove [41]. Bands attributed to β-N-glycosidic linkage (at 214 nm) and hydrogen bonding (224 nm) show no appreciable change in the complex spectra.

### Thermodynamic Profile of Nimustine-DNA Interaction

**Figure 8:**
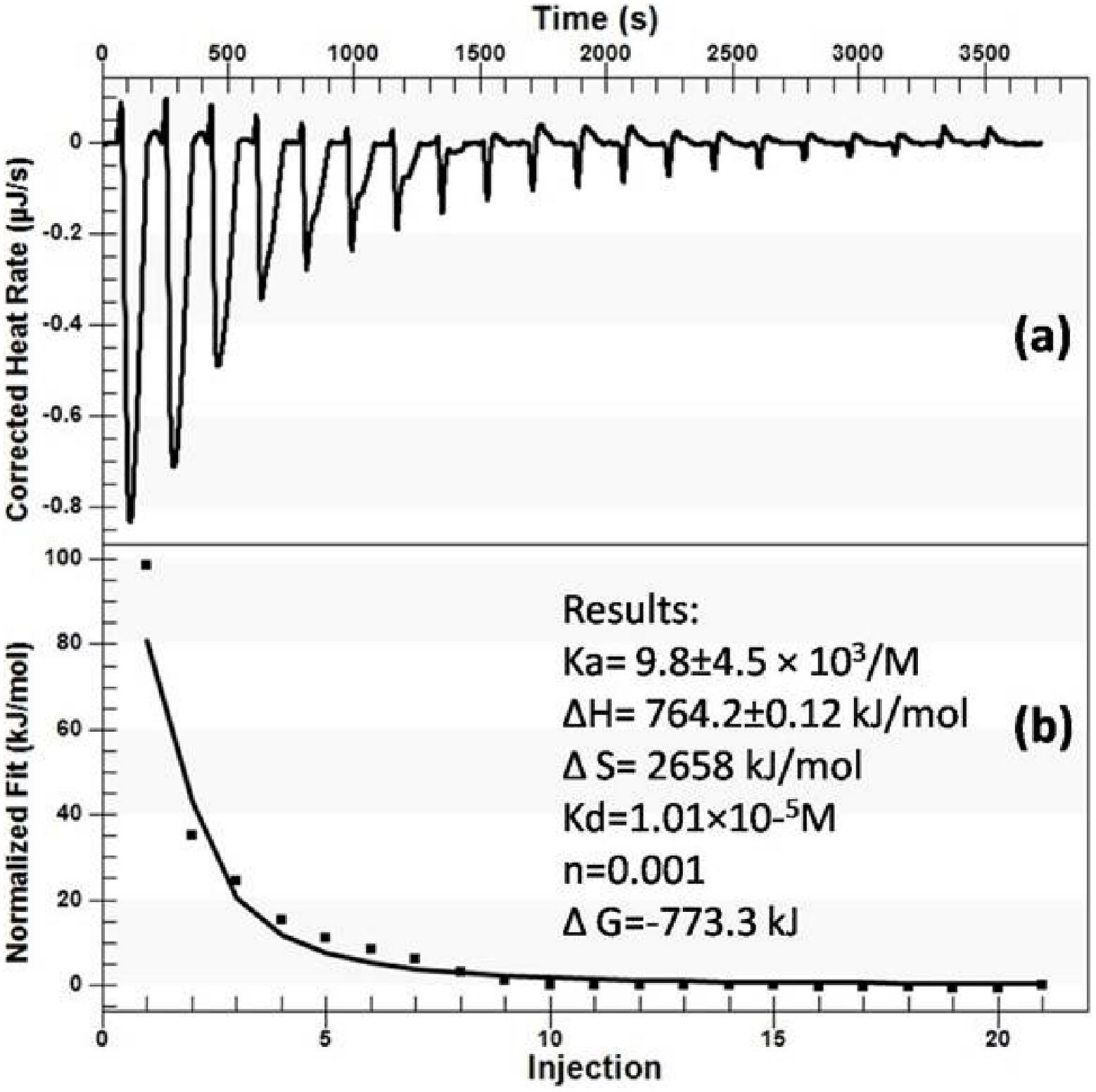
ITC curves for the binding of nimustine to DNA.

Figure 8 (a) shows the ITC titrations results, where each heat burst curve represents to a single nimustine injection to calf thymus DNA. The area below these curves is determined by integration to yield the associated injection heats (net injection heat). Net heat is then plotted against injection number depicted in the lower panel (Figure 8b). The dots reflect the experimental injection heat while the solid line represents the calculated fit of the data set. The data have been fitted to the single set of identical sites model that give a reasonable fitting of the experimental data. The interaction data represents entropy driven endothermic binding event with positive entropy change (ΔS) 2658 kJ/mol and positive enthalpy change (ΔH) 764.2±0.12 kJ/mol. The determined binding affinity constant (Ka) is found to be 9.8±4.5×10^3^/mole with Gibb’s free energy (ΔG) −773.3 kJ.

Nimustine-DNA interaction is favored by positive enthalpy changes that indicate groove binding of nimustine into double helix [42]. Furthermore, positive entropy suggests the disruption of the unique water molecules lining the DNA groove [19]. Reasonable explanation of this outcome may be the hydrophobic interaction of nimustine into major groove where it makes a stable contact with DNA to perform alkylation [19], [20]. This is in complete agreement with spectroscopic results.

## Conclusions

The spectroscopic results show that nimustine is a major groove directed alkylating agent. Further analysis illustrates that nimustine interaction occurs via guanine (C6 = O6) and thymine (C4 = O4) reactive sites located in DNA major groove. Some degree of external interaction with phosphate-sugar backbone has also been observed. CD spectral results suggest the formation of an intermediate form of DNA during the transition from B to C-form at local level after nimustine-DNA complex formation, although globally DNA remains in native B-form. Thermodynamically nimustine-DNA interaction is found to be entropy driven endothermic reaction. These findings may add to an understanding about the interaction mode of nimustine with DNA at molecular level.
